# Handling, Reproducing and Cryopreserving Five European Sea Urchins (Echinodermata, Klein, 1778) for Biodiversity Conservation Purposes

**DOI:** 10.3390/ani12223161

**Published:** 2022-11-16

**Authors:** Estefanía Paredes, Sara Campos, Alba Lago, Tracy Bueno, Julien Constensoux, Damian Costas

**Affiliations:** 1 Grupo ECOCOST, Centro de Investigación Mariña (CIM), Departamento de Ecoloxia e Bioloxía Animal, Universidade de Vigo, 36310 Vigo, Spain; 2 Universidad de Santiago de Compostela, 15075 Santiago, Spain; 3 Université de Montpellier, 163 rue Auguste Broussonnet, 34090 Montpellier, France; 4 Centro de Investigación Mariña (CIM), Universidade de Vigo, 36331 Vigo, Spain

**Keywords:** sea urchins, fertility, chilling injury, toxicity of cryoprotectants, cryopreservation

## Abstract

**Simple Summary:**

This is a basic study about the handling, reproduction and cryopreservation of cells from five different sea urchin species present in the Northeastern Atlantic Ocean: *Sphaerechinus granularis*, *Psammechinus miliaris*, *Echinus esculentus*, *Paracentrotus lividus* and *Echinocardium cordatum*.

**Abstract:**

In this work, five local sea urchin species found in European waters were studied. Four were regular species: *Sphaerechinus granularis*, *Psammechinus miliaris*, *Echinus esculentus* (Linnaeus, 1758) and the edible sea urchin *Paracentrotus lividus*; and one was an irregular species, *Echinocardium cordatum*. These five species of sea urchins have been studied regarding their fertility, toxicity of cryoprotecting agents, cryopreservation of different cell types and chilling injury. The baseline fertility is similar in *P. lividus*, *P. miliaris* and *S. granularis.* Nonetheless, the sperm:egg ratio, contact time and development of the fertilization envelope would need to be studied further on a case-by-case basis. Sperm can be maintained inactively in the gonad (4 °C), and oocytes also maintain quality in sea water (4 °C), even after 72 h. Sperm was cryopreserved for four species with some post-thaw intra specific variability, and embryo cryopreservation was only possible for *S. granularis*. Overall, this study provided a wider vision of the biology and reproduction of these species that will help us develop tools for their biodiversity conservation through cryopreservation.

## 1. Introduction

**Figure 1:**
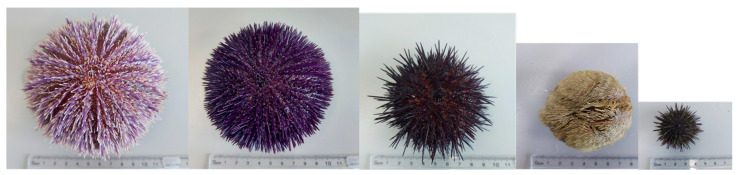
Sea urchin species, from left to right: *Echinus esculentus, Sphaerechinus granularis, Paracentrotus lividus*, *Echinocardium cordatum* and *Psamechinus miliaris* adults at scale.

There are more than 700 known sea urchin species widespread within the five oceans [1]. Galician local waters host at least 16 of those species, according to the inventory of [2] Echinoids in the Galician coasts (Supplementary Table S1). Of these, eleven are regular and five are irregular sea urchins (Figure 1).

In this work, five local species were studied. Four were regular (spiny, globular, live on rocky areas and present five-fold radial symmetry): *Sphaerechinus granularis* (Lamarck, 1816), *Psammechinus miliaris* (Müller, 1771), *Echinus esculentus* (Linnaeus, 1758) and the edible sea urchin *Paracentrotus lividus* (Lamarck, 1816), and one was irregular (soft spine, lives on sand or muddy bottoms and presents secondary bilateral symmetry); *Echinocardium cordatum* (Pennant, 1777). *Paracentrotus lividus* (Lamarck, 1816) is a regular sea urchin that can be easily found on benthic rock substrates or distributed in intertidal exposed or semi-exposed areas. *P. lividus* is a commercial species of economic importance, appreciated by the market for its gonads in a wide part of its distribution area in Europe [3,4]. It is possibly the best-known echinoderm, due to the multiple studies that have been carried out due to its economic, biological and ecological importance [5,6,7]. To date, several cryopreservation protocols have been developed for sperm [8,9] and embryos [10,11] of *P. lividus*.

*Sphaerechinus granularis* (Lamarck, 1816) is also a regular sea urchin with a globose body diameter of up to 10–15 cm. It can be shaded purple or white, and their spines are short and blunt. *S. granularis* is a subtidal species that frequently lives together with *Paracentrotus lividus* (on their subtidal end frequently found below 8 metres). It preferentially feeds on brown algae, but also coralline algae, seaweed leaves and their epiphytes or detritus [6]. Although it is edible, no information has been found about active fisheries for this species, although, historically, there have been punctual reports in Spain or France [12].

*Psammechinus miliaris* (P.L.S. Müller, 1771), a regular sea urchin with a small diameter, can reach 5 cm at adulthood. It is covered in short, equi-length, robust spines, and presents a greenish test and pale colored spines with purple tips. It can be found in intertidal areas at up to a depth of 100 m. *P. miliaris* distribution spans from the eastern Atlantic Ocean from Scandinavia to Morocco, but not in the Mediterranean Sea. The larvae often settle onto man-made structures, such as ropes, in aquaculture facilities. There are no active fisheries registered, although the idea has been explored [13].

*Echinus esculentus* (Linnaeus, 1758), is a regular sea urchin with a body globose, pointed, covered by strong and grouped short barbs, forming a uniform cover of light pink color. *E. esculentus* can easily measure 10 cm in diameter; it and *S. granularis* are the largest species on the Galician coasts [6]. It lives on rocky bottoms, often covered by laminaria, up to a depth of 15 m. There are active fisheries in Iceland and Ireland [14]. This species is listed as near threatened by the IUCN, although it has not been evaluated in Europe (EUNIS 2020).

*Echinocardium cordatum* (Pennant, 1777), is an irregular sea urchin with a diameter of up to 9 cm, with small yellowish flexible spines. *E. cordatum* is found in sandy beds (both muddy and coarse sand) at depths of up to 175 m. It is very frequent in the subtidal sandy beds that extend throughout the coastal strip of Galicia [6]. Buried in the sand, it feeds by ingesting detrital sediments [15]. There are no reports of irregular sea urchins which been extracted for human food, although in principle, they are edible.

Although some species has been identified as good candidates for future commercial extraction [16,17,18,19,20] little is known about their biology, population dynamics, abundances and reproduction or growth rate due to their low commercial interest until now. In addition, these species can be found near or within *P. lividus* exploitation areas and might, therefore, be impacted by the activity of the fishery. 

In situ conservation of species can be reinforced with ex situ conservation methods, such as cryopreservation and biobanking. In order to preserve genetic diversity and biodiversity, it is crucial to sustaining healthy ecosystems and preserving their capacity for adaptation [21]. In order to achieve this, selective breeding can ensure genetic variability during repopulation efforts, and cryopreservation can provide a wide source of genetically diverse material from biobanks. Biobanks can also be used to enhance the extent and quality of knowledge on certain species by providing easy access to marine biological resources with which scientists can work [22,23].

Out of the all the sea urchin species known, only 10 of those species have been studied in cryobiology, and not all have successfully developed cryopreservation protocols [24]. Despite this, only one species *Paracentros lividus*, has had a developed and functional cryopreservation protocol for sperm and embryos until now (Supplementary Table S2).

One of the key parameters fur a successful cryopreservation is understanding the sensitivity of the species and cells to different cryoprotecting agents (CPAs). The role of these chemical compounds is to protect the cells during cooling by depressing the freezing point of the solutions, in addition to reducing the possibility of large ice crystals forming during cooling. Several chemicals have been identified as having cryoprotective action, such as dimethyl sulfoxide (Me_2_SO), propylene glycol (PG), ethylene glycol (EG), glycerol (GLY), methanol (MetOH), sucrose (SUC) and other sugars or polyvinylpyrrolidone (PVP). However, it is necessary to understand the concentrations needed and their effects on cells [25,26].

The aim of this work was to boost the knowledge of reproduction and cryopreservation of sea urchin species by developing suitable protocols for different cell types. It is expected that this study will encourage aquaculturists, fishery managers and researchers to explore the benefits of cryopreservation and improve their knowledge as well as the biodiversity conservation of these ecologically important organisms.

## 2. Methods

### 2.1. Collection of Biological Material

The sea urchin species were the following: *Paracentrotus lividus*, *Sphaerechinus granularis*, *Psammechinus miliaris*, *Echinus esculentus* and *Echinocardium cordatum*. They were collected by scuba diving in the Cies islands at the Illas Atlanticas de Galicia National Park. After collection, these sea urchins were maintained at the Estacion de Ciencias Mariñas de Toralla (ECIMAT) from Centro de Investigación Mariña (CIM)-Universidade de Vigo. All of the required permits, both from the local government and from the National Park Authorities, are effective for the animal collection, and another permit for macroalgae collection for sea urchin feeding was obtained from the local government.

### 2.2. Handling of Sea Urchin Gametes and Fertilization

The standardized protocol for *P. lividus* handling was as follows: collection and transportation from sampling site to the lab in short periods of time (less than 1 h, whenever possible), and covering the animals with seaweed in order to keep them moist and cool during transport. Whenever possible, sea urchins were differentiated by sex as soon as arriving to the lab, using of [27] Paredes and Costas 2020 methodology. Different sexed sea urchins were stored separately in tanks with running seawater at the same temperature recorded at the collection point.

Gametes were obtained by dissection of the sea urchins and direct aspiration from the gonads. In all cases, gamete quality was examined prior experimentation; in males, sperm should be active when mixed with sea water [27]. In females, oocytes should be round and homogeneous in color. Whenever possible, the percentage of fertilization was used as a quality assessment criterion as well.

### 2.3. Setting up a Baseline for the Species Fertility

The baselines of natural fertility for each species were established in local sampled populations. Taking as a referent our standard fertilization procedure for *P. lividus*, fertilization was carried out with a 10–20 sperm:egg ratio at a density of 40 egg/mL, with a contact time of 15 min. The percentage of fertilization was assessed afterwards by counting the cells showing a fertilization membrane (n = 100). Embryos were allowed to develop for 48 h in filtered sea water (FSW) until a 4-arm pluteus larvae stage was achieved, and then the percentage of normal developed larvae was assessed (n = 100). Experiments were conducted in triplicate.

### 2.4. Sperm and Oocyte Viability with Low Temperature Storage

Sperm and oocytes from all species were kept at 4 °C and 18 °C for several hours (1, 2, 3, 4 and 72 h post dissection), either inactivated inside the gonad or activated in sea water. Once this time had passed, a subsample of oocytes and sperm were evaluated in order to understand how long the gametes would remain viable once the animal had been dissected, as well as whether low temperatures could extend the fertility. Viability was measured by checking motility and percentage of egg fertilization compared to the controls at the time of the dissection.

### 2.5. Toxicity of Cryoprotecting Agents (CPAs)

The toxicity of the cryoprotective agents Dimethyl sulfoxide (Me_2_SO), Ethylene glycol (EG), Propylene glycol (PG), Glycerol (GLY), Methanol (MeOH), N,N-dimethylformamide (DMF), Trehalose (TRE), Fructose (FRU), Sucrose (SUC) and Polyvinylpyrrolidone (PVP) was evaluated for the cell types as well as two species, *P. lividus* and *S. granularis*, using one male and one female for each fertilization trial conducted. The cells from one pair of sea urchins [28] were exposed to CPAs stepwise in order to avoid/minimize osmotic shock. After equilibration, the CPA was diluted stepwise with filtered sea water (FSW, 0.22 µm + UVA), following guidelines by Paredes and Bellas (2009). Treatments were then fixed with formalin and examined under a microscope in order to determine the fertilization of the eggs (when studying Sperm after 15 min contact time) or the percentage of normal 4-arm pluteus larvae (n = 100), as well as the average larval growth (n = 35), when working with embryos (after 48 h of incubation at 18 °C). Whenever possible, the NOEC (No Observed Effect Concentration) and LOEC (Lowest Observed Effect Concentration) were determined by calculating the significant differences in larvae size between treatments and control after 48 h, and taking into account larval normal development, following [26,29,30]. Quality control of the gametes has been conducted according to [31].

### 2.6. Cryopreservation Methods

Sperm cryopreservation was performed using liquid nitrogen vapor after 5 min of equilibration, with a 15% Me_2_SO (*v/v*) final concentration in sea water at room temperature (20 °C). After mixing with cryoprotectants, sperm samples were packed in 0.25 mL straws and cooled on a floating rack of 5 cm (cooling rate: 11 °C/min) over the liquid nitrogen level on a closed Styrofoam box for 8 min. Samples were then plunged into liquid nitrogen for storage. Straws were later thawed by immersion in a 35 °C water bath for 6 s. The cooling rate at 5 cm was calculated, by use of a thermocouple recording, to be 20 °C/min.

Sea urchin embryos were cryopreserved by use of a controlled rate freezer (Cryologic, Australia Lt., Blackburn, Australia) using 2 mL vials and a protocol that had been developed for *P. lividus* early blastulas [11]. Cryoprotecting agents were added stepwise in 15 equimolar steps of 1 min, and cooled down at a rate of 1 °C/min [10]. Vials were thawed by 2-min immersion in a 18 °C water bath until the ice melted. Then, cryoprotecting agents were diluted by adding filtered sea water in 12 equimolar steps of 1 min. Cells were then rinsed with FSW and incubated for 2 to 3 days until 4 arm-pluteus larvae were obtained, and they were then fixed with formalin for further analysis. Embryo cryopreservation was tested for all five species using the exact protocol developed for *P. lividus*. One male and one female from each species were used to obtain healthy embryos for each cryopreservation trial. 

### 2.7. Cooling and Warming Rate Calculations

In the case of vapour cooling, the actual cooling rate (in °C/min) was calculated by measuring the temperature inside a straw filled with FSW, using a thermocouple. The straw was placed on top of the floating rack (5 cm above the liquid nitrogen surface) for 15 min. Then, the straws were quickly transferred to liquid nitrogen for storage. The cooling rates were calculated from a linear regression of temperature vs. time (data available in Supplementary, Figure S1) and compared with data from the literature. Warming rates were sometimes too fast for our thermocouple sensor, and were, therefore, as extracted from the literature, 2500 °C/min for a straw [32] and 6 °C/min for 2 mL vials in an 18 °C water bath [33]. This measurement helped to optimize the cooling time of the protocol for the maximum cooling rate.

### 2.8. Statistics

The statistical analysis was conducted using a one-way ANOVA test, as well as Bonferronni and Dunnet post hocs with SPSS^®^ 15.0.1 (following the [34,35] Newman (1995) and Sokal and Rohlf (1995) method). The raw data (percentages) were normalized with an angular transformation when possible. Otherwise, we used a Kruskal–Wallis non-parametrical test with a Conover–Inman post hoc instead. 

## 3. Results 

### 3.1. Baseline Fertility and Viability with Temperature of the Gametes

**Figure 2:**
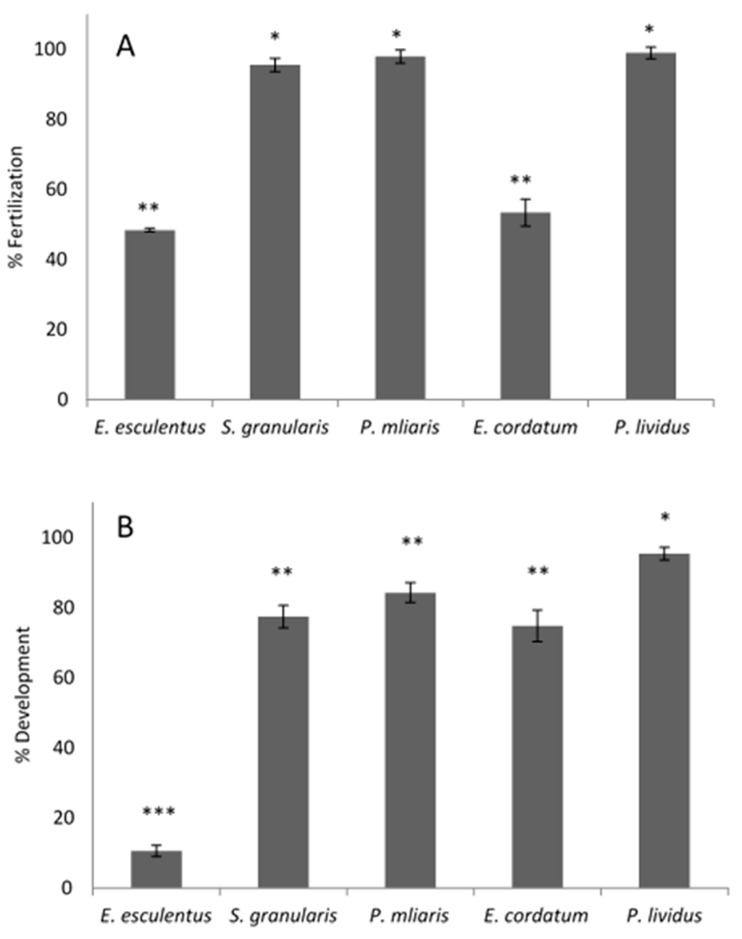
Percentage of fertilization (**A**) and development to 4-arm pluteus larvae (**B**) for each species (n = 100) (mean% ± SD). Asterisks mark significant differences among species (*p* ≤ 0.05).

The baseline fertility results are shown in Figure 2A. High interspecific variability in fertility was obtained when using the criteria for *P. lividus*, which quantified the presence/absence of the fertilization membrane 15 min after the mixing of sperm and oocytes (10/20:1 ratio). In the case *of S. granularis, P. miliaris and P. lividus*, results were similar, and the only significant differences (*p* ≤ 0.05) were found with *E. esculentus* and *E. cordatum*. In Figure 2B, corresponding embryo development was obtained from the prior fertilization. Again, *E. esculentus* and *E. cordatum* showed a different result than the other three species; in the case of *E. esculentus*, the number of developed larvae were lower than expected according to the fertilization observed in Figure 2A. On the other hand, *E. cordatum* shows more larvae developed after incubation than expected according to the fertilization observed in Figure 2A.

Percentage of fertilization was not affected for the first 4 h after dissection in any of the treatments for *P. lividus*, *P. miliaris* or *S. granularis*. However, *E. esculentus* have shown lower fertilization percentages in the first hours, followed by increased fertilization percentages in the next 3 h (Figure 2). After 72 h, the gametes of *P. lividus*, *P. miliaris* and *S. granularis,* stored at 4 °C sperm in gonad with 4 °C oocyte and 4 °C activated sperm with 4 °C oocyte, were perfectly viable. *E. esculentus* has shown viability for the above treatments, as well as when using 18 °C activated sperm with 4 °C oocytes. All data can be examined in detail in the supplementary data, Table S3.

**Figure 3:**
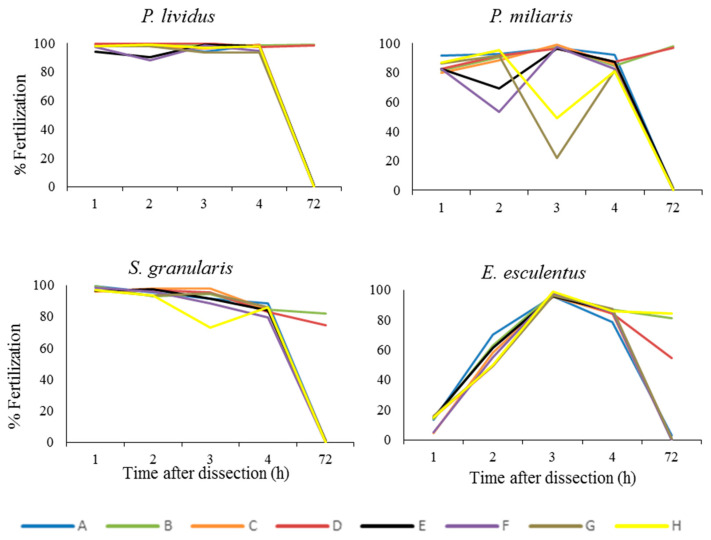
Percentage of fertilization after storage of the gametes at room temperature (18 °C) and fridge temperature (4 °C) for four species of sea urchins. The treatment combinations used to observe fertilization after 1, 2, 3, 4 and 72 h of keeping the gametes at 4 °C and 18 °C were the following: (A) 4 °C sperm in the gonad with 18 °C oocyte; (B) 4 °C sperm in gonad with 4 °C oocyte; (C) 4 °C activated sperm with 18°C oocyte; (D) 4°C activated sperm with 4 °C oocyte; (E) 18 °C sperm in the gonad with 18 °C oocyte; (F) 18 °C sperm in the gonad with 4 °C oocyte; (G) 18 °C activated sperm with 18 °C oocyte; (H) 18 °C activated sperm with 4 °C oocyte.

In general, maintaining the inactive sperm in the gonad in the fridge (4 °C) is an effective way to extend their viability. Oocytes also maintain their quality for a longer period of time at this temperature in sea water, even 72 h post-dissection (Figure 3, data available in Supplementary Table S1).

### 3.2. Toxicity of CPAs 

The toxicity of several cryoprotecting agents (CPAs) for different cell types and the calculated NOEC and LOEC are listed in Table 1 for *P. lividus* and in Table 2 for *S. granularis.* A general increase was found in tolerance to CPA exposure with development stages, oocytes being the most sensitive cell types and larvae being the most resistant for both species.

When comparing *P. lividus* and *S. granularis*, the latter is more sensitive to CPAs. In several cases, we were not capable of calculating the NOEC levels (n/a in Table 2) as all the concentrations tested already had significant toxic effects. When analyzing toxicity by CPA type, in general, the toxicity of non-permeant CPAs such as PVP or sugars was lower than permeating CPAs. Among permeating CPAs, Me_2_SO, PG and Gly were the most toxic, and EG and Meth were lower.

### 3.3. Sperm Cryopreservation

**Figure 4:**
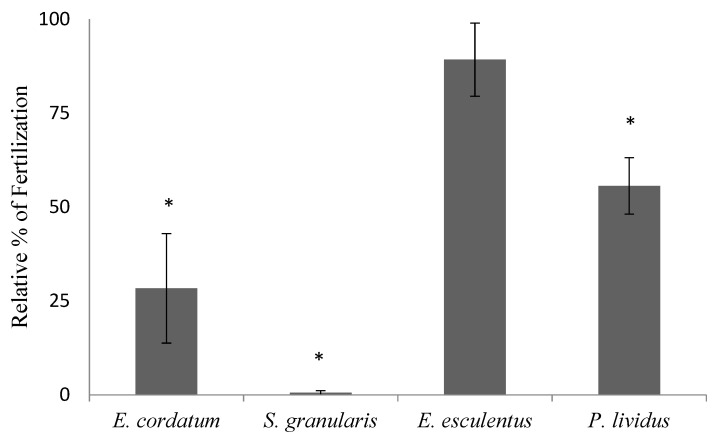
Relative percentage of fertilization, mean ± SD, obtained with sperm that had been cryopreserved using 1.5 M Me_2_SO for four species of sea urchins (n = 3 individuals), using a floating rack at 5 cm above liquid nitrogen. Sperm to egg ratio was constant at 10–20:1, and contact time allowed was 15 min before the fertilization assessment. (Controls showed 60 ± 10%, 35 ± 5.5% (unusually low), 95 ± 3% and 98 ± 2.1% fertilization, respectively). Asterisks show significant differences with their specific controls (*p* ≤ 0.05).

For the long-term conservation of sperm, we have studied the transference of the same cryopreservation protocol among species (Figure 4). The fertilization capacity of the post-thaw sperm was significantly different (*p* ≤ 0.05) than fresh controls (fresh sperm showed quite high variability in quality as well, especially notable in *S. granularis*) in all species except *E. esculentus.* In all cases, motility of the sperm cells was seriously affected, but the cells retained plasma membrane integrity (as tested by fluorescence using live/dead staining). 

### 3.4. Embryo Cryopreservation of S. granularis

**Figure 5:**
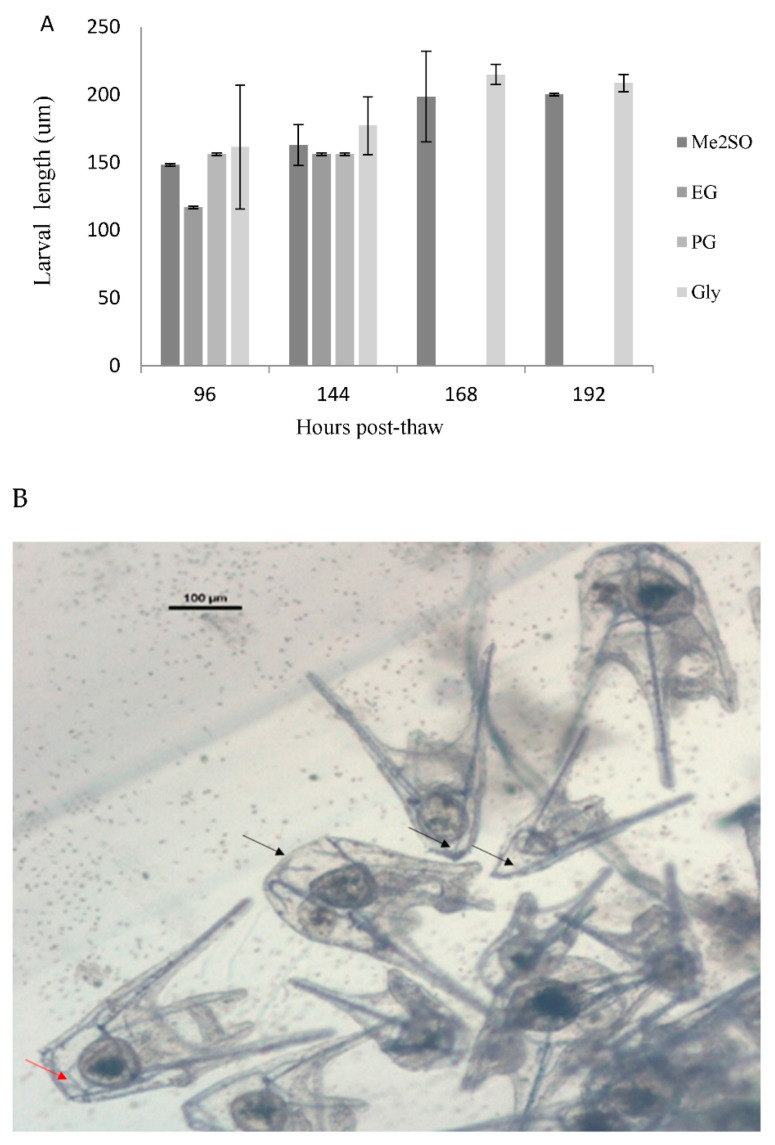
(**A**) Cryopreserved blastula embryos of *S. granularis* larval length after incubation in seawater after different incubation times (from 96 to 192 h at 18 °C) when using Paredes and Bellas’ 2014 cryopreservation protocol and 1.5 M of different CPAs. (**B**) Picture shows larvae obtained after 192 h of incubation of blastulas and cryopreserved with 1.5 M Gly. Red arrows show normal skeletogenesis, and black arrows point out frequent abnormalities.

Embryo cryopreservation has been previously reported only in *S. intermedius* and *P. lividus* (Supplementary Table S2), and long-term viability of those embryos has been detailed for *P. lividus* [11]. We have transferred their protocol *to S. granularis* larvae and obtained over 50% post-thaw survival. Embryos were able to successfully metamorphose to larvae and grow when using 1.5 M Me_2_SO. After a detailed toxicity study of the CPAs (Table 2), where EG showed promising low toxicity as a CPA, the results of cryodamage were, nonetheless, surprisingly decoupled from toxicity. Despite being quite toxic, 1.5 M Glycerol protected the cells from cryodamage and produced the same survival as Me_2_SO (also quite toxic, Table 2, Figure 5A), and the larvae developed quite normally (although they remained significantly smaller than control larvae for their age at all times). Still, we have detected some skeletal damage and anomalies that need to be studied further (Figure 5B). This was the first attempt to cryopreserve embryos of this species.

## 4. Discussion and Conclusions

Among the five sea urchin species, some are more closely related. For example, *P. lividus, E. esculentus* and *P. miliaris* belong to the family Echinidae, while *E. cordatum* is from the Loveniidae and *S. granularis* is from the Toxopneustidae family [1].

The study of the fertility of these five species of sea urchin allowed us to understand the baseline fertility, and also helped us to optimize and adapt the protocols in order to maximize the fertilization success. The fertilization study (Figure 2) gave us insight on how to assess fertilization, in the case of *P. lividus*. The time that it takes from fertilization to the lifting of the fertilization membrane (which is our visual cue to know the egg has been fertilized) is a matter of seconds. Nonetheless, we left the egg/sperm in contact for 15 min before calculating the percentage of fertilization of fresh gametes, which reached 90%. One of the main conclusions that we could extract from the data in Figure 2 and Figure 3 was that in *E. esculentus,* the baseline fertilization of fresh gametes after those 15 min was only 48%, and the rate of successfully developed larvae was even lower (Figure 2). However, when monitoring fertilization over time, it seems that we could obtain a high percentage of fertilization (Figure 2, 98% fertilization after 3 h). The capacitation of the sperm of this species is complex; this effect is clear even in non-activated sperm preserved in the gonad at 4°C. Simultaneously, we may need to allow a longer sperm/egg contact time (this seems to be the case for *E. cordatum,* as we registered only 53% fertilization, but found more than 70% development after 48 h) or a higher sperm to egg ratio. In the future, these questions might be solved with the use of computer-assisted sperm analysis (CASA) to evaluate sperm motility as well as by testing increasing contact times and optimizing the sperm:egg ratio for each species.

We do not have all the data to compare all the sea urchins (as their breeding seasons only overlap slightly). For example, there were toxicity data for only two species in this work. In general, sea urchins, even those in the same family, do not show the same pattern of response to toxicity, nor regarding fertility or response to a complex process such as cryopreservation. When transferring the embryo cryopreservation protocol for *P. lividus* blastulas to other species [10], results were only obtained with *S. granularis,* and no success was obtained with *E. esculentus* (which is a closer relative to *P. lividus*). This is also the case when cryopreserving sea urchin sperm; again, significant differences were found in post-thaw sperm fertility in the same conditions. In this case, the protocol designed for *P. lividus* worked even better on *E. esculentus*. Our hypothesis was that the differences were due to the different sensitivity to cryoprotectants of these species (as could be seen in Table 1 and Table 2). Sea urchins are generally sensitive to chemicals, and that is why they are commonly used as model organisms in the evaluation of marine water quality [36]. As cryoprotecting agents are chemicals, many are cell-permeating. Sea urchin cells do show high sensitivity to the presence of methanol or dimethyl sulfoxide in the water. This is in agreement with prior data on sea urchin cryopreservation [26,29]. The data highlight the idea that the later the development stage, the more resistant they are to the presence of the cryoprotectants, which agrees with the general principle of ecotoxicology that earlier development stages are more sensitive to chemicals present in the water [36,37]. This has also been observed in the cryopreservation of other marine species, such as mollusks [29,38]. 

Cryopreservation protocols for sea urchins need to be specifically designed, starting by conducting a toxicity test of the intended cryoprotecting agents. Nonetheless, the information that can be extracted from crossing the toxicity data and the outcome of embryo cryopreservation (Table 2, Figure 4) is that low toxicity of a cryoprotectant is desirable, but not always determinant. The data showed that the lowest toxicity for *S. granularis* blastulas was EG. Nonetheless, the survival obtained was zero, and higher survival was obtained with Me_2_SO or Gly as cryoprotectants, which presented higher toxicity. This has been found in *P. lividus* [10,26], when Me_2_SO showed successful results despite being one of the most toxic compounds for the species. The rationale for this counterintuitive phenomenon is that there needs to be a balance between low toxicity of the chemical and high capacity of cryoprotection (for example, characteristics such as good permeability in the cells or fast permeation in all cell compartments that allows for the protection of all cells) [39]. Unfortunately, the data regarding the permeability of cryoprotectants for sea urchin cells have only been described for the *Evechinus chloroticus* eggs [40], and, therefore, factors such as equilibration time, which will allow the CPAs to maximize cryoprotection, cannot be adjusted, only approximated experimentally.

Finally, it is very interesting to notice that each species has a different response to chilling injury (injury due to low temperature exposure over 0 °C). In general, it has been argued that marine invertebrate eggs are sensitive to chilling. However, in this study, sea urchin eggs could be fertilized after being exposed to 4 °C for over 72 h, with an average fertilization rate around 80%, regardless of the species. The sperm of *P. lividus, P. miliaris* and *S. granularis* also maintains its functionality after storage at 4 °C, either inactive in the gonad or activated. For *E. esculentus,* fertility was retained after activation and storage in sea water at 18 °C. This disagrees with what we have known so far about chilling injuries in sea urchins [41,42], and whether chilling injury is dependent on the composition of the eggs, which may vary depending on the season or the geographical area, should be explored. This has been the first attempt to study chilling injuries across five different species of sea urchins and no long-term developmental studies have been conducted to assess the complete effect.

These five species of sea urchins distributed in European waters have been studied regarding their fertility, sensitivity to cryoprotecting agents, cryopreservation of different cell types and chilling injury. This provides a wider vision, although partial, of their reproduction, which will help us to develop tools for their conservation. More research is needed in order to deepen the knowledge, adapt the protocols for optimization and understand processes such as cryoprotecting agent toxicity or chilling injury in sea urchin cell types.

## Figures and Tables

**Table 1:** Cryoprotecting agents (CPA) toxicity as “No Observed Effective Concentration” (NOEC) and “Lowest Observed Effect Concentration” (LOEC) for 4 cell types of *Paracentrotus lividus,* following the methodology by [26].

CPA	NOEC (M)				LOEC (M)			
	Oocyte	Fert. Oocyte	Blastula	Pluteus Larvae	Oocyte	Fert. Oocyte	Blastula	Pluteus Larvae
Me_2_SO	0.5	1	1.5	2	1	1.5	2	2.5
EG	1	1	1	0.5	1.5	1.5	1.5	1
PG	0.68	0.68	1.36	0.5	1.36	1.36	2.04	1
GLY	–	–	–	0.5	–	–	–	1
Meth	2.5	–	–	1	3	–	–	1.5
DMF	2	–	–	–	2.5	–	–	–
TRE	0.05	0.05	0.05	–	n/a	n/a	n/a	–
PVP	0.75	0.75	0.75	–	n/a	n/a	n/a	–
CPA cocktails	NOEC (M)				LOEC (M)			
ME_2_SO + TRE	1	1	1	–	1.5	1.5	1.5	–
ME_2_SO + PVP	n/a	n/a	n/a	–	1	1	1	–
EG + TRE	1	1	1	–	1.5	1.5	n/a	–
EG + PVP	2	2	2	–	n/a	n/a	n/a	–
PG + TRE	2.04	2.04	2.04	–	n/a	n/a	n/a	–
PG + PVP	2.04	2.04	2.04	–	n/a	n/a	n/a	–

Cryoprotecting agent acronyms: Me_2_SO: dimethyl sulfoxide, EG: ethylene glycol, PG: propylene glycol, Meth: methanol, GLY: glycerol, DMF: dimethyl formamide, TRE: trehalose and PVP: polyvinyl pyrrolidone. N/a indicates no data available because the concentration falls out of our experimental range, and (–) indicates never tested.

**Table 2:** Results of cryoprotecting agents (CPA) toxicity as No Observed Effective Concentration (NOEC) and Lowest Observed Effect Concentration (LOEC) for 2 cell types of *Sphaerechinus granularis*, following the methodology by [26].

CPA	NOEC (M)		LOEC (M)	
	Oocyte	Blastula	Oocyte	Blastula
Me_2_SO	n/a	n/a	0.5	0.5
EG	n/a	2	0.5	3
PG	n/a	n/a	0.5	0.5
GLY	n/a	n/a	0.5	0.5
Meth	0.5	1	1	1.5
TRE	0.03	0.04	0.04	0.05
PVP	n/a	n/a	0.25	0.25
SUC	n/a	n/a	0.25	0.25
FRUC	n/a	n/a	0.25	0.25
GLU	n/a	a/a	0.25	0.25

Cryoprotecting agent acronyms: Me_2_SO: dimethyl sulfoxide, EG: ethylene glycol, PG: propylene glycol, Meth: methanol, GLY: glycerol, TRE: trehalose, PVP: polyvinyl pyrrolidone, SUC: sucrose, FRUC: fructose and GLU: glucose. N/a indicates no data available because the concentration falls out of our experimental range.

## Data Availability

Data will made available upon request.

## Supplementary Materials

The following supporting information can be downloaded at: https://www.mdpi.com/article/10.3390/ani12223161/s1, Figure S1: Cooling rate calculation; Table S1: List of sea urchins catalogued in Galician waters; Table S2: Updated list of cryopreservation studies published on Sea urchins [43,44,45,46,47,48,49,50,51,52,53,54,55,56]; Table S3: Fertilization Data Temperature storage experiment.
