# Supplementary material for: Handling, Reproducing and Cryopreserving Five European Sea Urchins (Echinodermata, Klein, 1778) for Biodiversity Conservation Purposes
Source: Animals (Basel). 2022 Nov 16;12(22):3161. doi: 10.3390/ani12223161 (PMC9686528; doi:10.3390/ani12223161)
Supplement: Supplementary file 1 [file animals-12-03161-s001.zip › animals-1974580-supplementary.pdf]

Supplementary material

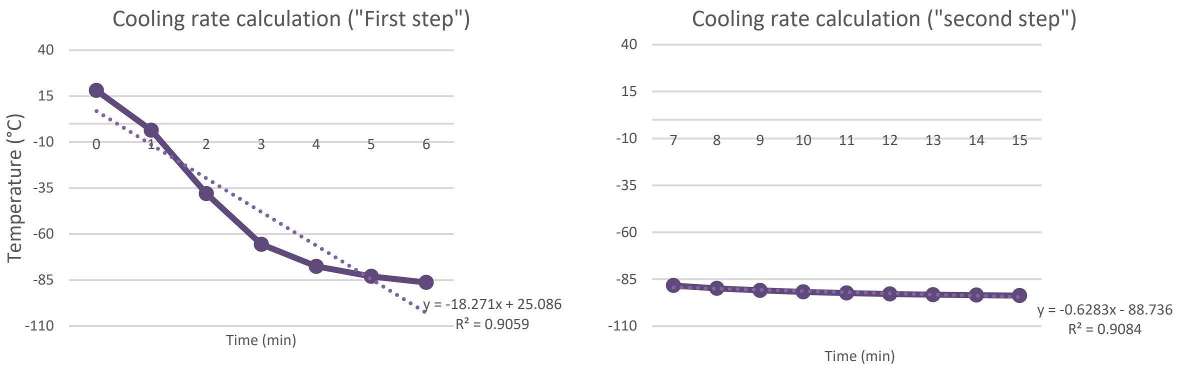

Supplementary Figure S1. Cooling rates were monitored using a thermocouple by registering the real temperature inside of a straw at the 5 cm floating rack per minute by triplicate from room temperature (18ºC) to -80ºC and the data has been represented in two graphs due to the change in cooling rates along time.

Supplementary Table S1. List of sea urchins catalogued in Galician waters [2,6].

Regular/ Irregular	Order	Family	Species	WORMS ID
Regular	Cidaroida	Cidaridae	*Cidaris cidaris*	124257
			Stereocidaris ingolfiana	124267
	Echinomurioida	Echinothurida	*Araeosoma fenestratum*	149880
			Phormosoma placenta	124343
	Arbacioida	Arbaciidae	*Arbacia lixula*	124249
	Temnopleurida	Toxopneustidae	*Sphaerechinus granularis*	124427
	Echinoida	Echinidae	*Echinus acutus*	124277
			Echinus alexandri	124280
			Echinus esculentus	124287
			Paracentrotus lividus	124316
			Psammechinus miliaris	124319
Irregular	Clypeasteroidea	Fibulariidae	*Echinocyamus pusillus*	124373
	Cassiduloida	Cassiduloidae	*Neolampas rostellata*	144254
	Spatangoidea	Brissidae	*Brissopsis lyrifera*	124373
		Spatangidae	*Spatangus purpureus*	124418
		Loveniidae	*Echinocardium cordatum*	124392

Supplementary Table S2. Updated list of cryopreservation studies published on Sea urchins sperm, oocytes, embryos and larvae from 1960 to 2021. The highest survival results reported in each study were listed. Cryoprotecting agents (CPAs) acronyms: Me_2SO: Dimethyl sulfoxide, EG: Ethylene glycol, PG: Propylene glycol, TRE: Trehalose.

Species	Cell	Refference	Cryopreservation Protocol	Survival Assessment
Hemicentrotus pulcherrimus	Embryos	[43, 44]	Me_2SO 1.5M. Cooling rates 10-40°C min^{-1}. Thawing in air 15°C min^{-1}	10% development to larvae
	Larvae	[45]	Me_2SO 1M. Cooling rates 10-40°C min^{-1}. Thawing in air 15°C min^{-1}	90% survival
Strongylocentrotus nudus	Larvae	[44]	EG 1.5M. Cooling rate 10°C min^{-1}.Thawing rate 7°C min^{-1}	90% active swimming
Strongylocentrotus intermedius	Embryo	[43, 44]	EG 1.5M. Cooling rate 10°C min^{-1}. Thawing rate 7°C min^{-1}	90% active swimming
		[46]	Me_2SO 1-1.5M. Cooling rate 6-8°C min^{-1}. Thawing in water bath 19°C	≥90% survival
		[47]	Me_2SO 1.5M. Acording to [46]	0.1-0.2% development to 2nd generation
		[48]	Me_2SO 1M + 1 mg ml^{-1} Antioxidant. Acording to [46]	60% swimming post-thaw, 1% develop.
		[49]	Me_2SO 6% (v/v)+ 40 mM TRE + 0.15% (w/v) Antiox. Cooling rate 7°C min^{-1}. Thaw in water bath 10-15°C	40% survival
	Larvae	[44]	EG 1.5M. Cooling rate 10°C min^{-1}. Thawing rate 7°C min^{-1}	90% development
		[48]	Me_2SO 1M + 1 mg ml^{-1} Antiox., Acording to [46]	20% active swimming
Anthocidaris crassispina	Sperm	[50]	According to [56]	10% motility
Loxechinus albus	Larvae	[51]	Me_2SO 1M. Two step cooling rate 3:10 °C min^{-1}. Thawing in water bath 15°C 30 s.	77% survival after 24 h., 55% survival after 21 d.
Tetrapigus niger	Sperm	[51, 52]	Me_2SO 1.2M. Two step cooling rate 6:25 °C min^{-1}. Thawing in water bath 17°C 12 s.	96% fertilization, 56% development after 24h.
Strongylocentrotus droebachiensis	Sperm	[53]	Me_2SO 12% (v/v).Cooling rate 5°C min^{-1}. Thawing at room temperature 45 min.	motility score 4 of 10
Evechinus chloroticus	Sperm	[54]	Me_2SO 2.5-7.5% (v/v). Cooling rate 50°C min^{-1}. Thawing in water bath 15°C 30 sec.	95% fertilization

	Larvae	[55]	Me_2SO 1.5M. Cooling rate 2.5°C min^{-1}. Thawing in water bath 15°C 30 s.	91% motility
Paracentrotus lividus	Sperm	[8]<br><br>Current study	Me_2SO 7% (v/v). Cooling rate 20°C min^{-1}. Thawing rate 15°C min^{-1}<br><br>Me_2SO 15% (v/v). Cooling over LN2 vapour (5cm, 8 min.), Thawing in water bath 35ºC 6 sec.	90% motility, 50% normal larvae<br><br>55.6% fertilization
	Oocytes	[23]	Me_2SO NOEC:0.5M, EG NOEC:1M, PG NOEC:0.68M	Toxicity tests of CPAs
	Embryos	[10,11]	Me_2SO 1.5M + 0.04M TRE. 1°C min^{-1}. Thawing in water bath 18°C	50-80% normal larvae after 96 h.
Pseudocentrotus depressus	Sperm	[56]	Me_2SO 10% (v/v). Cooling rate 6°C min^{-1}. Thawing rate 10-15°C min-1	13-33% fertilization
Echinus esculentus	Sperm	Current study	Me_2SO 15% (v/v). Cooling over LN2 vapour (5cm, 8 min.), Thawing in water bath 35ºC 6 sec.	89.2% fertilization
Sphaerechinus granularis	Sperm<br><br>Embryos	Current study	Me_2SO 15% (v/v). Cooling over LN2 vapour (5cm, 8 min.), Thawing in water bath 35ºC 6 sec.Me_2SO 1.5M + 0.04M<br><br>TRE. 1°C min^{-1}. Thawing in water bath 18°C	1% fertilization, cells alive not movement<br><br>50% survival post-taw
Echinocardium cordatum	Spem	Current study	Me_2SO 15% (v/v). Cooling over LN2 vapour (5cm, 8 min.), Thawing in water bath 35ºC 6 sec.	28.4% fertilization

Supplementary Table S3. Average (%) fertilization after keeping the gametes at different temperatures along 72 hours. Treatments are, (1) 4 °C sperm in gonad + 18 °C oocyte; (2) 4 °C sperm in gonad + 4 °C oocyte; (3) 4 °C activated sperm + 18 °C oocyte; (4) 4 °C activated sperm + 4 °C oocyte; (5) 18 °C sperm in gonad + 18 °Coocyte; (6) 18 °C sperm in gonad + 4 °C oocyte; (7) 18 °C activated sperm + 18 °C oocyte; (8) 18 °C activated sperm + 4 °C oocyte.

		Paracentrotus lividus			*Psammechinus miliaris*			*Sphaerechinus granularis*			*Echinus esculentus*		
**Time (h)**	**Treatments**	**Average (%)**	**Desv. St**	**error st**	**Average (%)**	**Desv. St**	**error st**	**Average (%)**	**Desv. St**	**error st**	**Average (%)**	**Desv. St**	**error st**
1	1	98.0	2.0	0.82	91.8	2.1	0.83	99.3	0.5	0.41	13.3	5.9	1.40
	2	99.3	1.0	0.56	81.5	9.3	1.76	99.3	1.0	0.56	13.8	6.2	1.44
	3	98.3	1.0	0.56	80.0	8.2	1.65	96.0	1.6	0.74	4.5	3.0	1.00
	4	99.5	0.6	0.44	82.3	6.3	1.45	96.5	2.1	0.83	15.0	3.7	1.12
	5	94.3	5.9	1.40	82.5	9.8	1.81	96.5	3.4	1.07	15.0	4.4	1.21
	6	97.3	2.5	0.91	82.3	5.3	1.33	98.3	2.1	0.83	4.8	3.9	1.13
	7	98.0	1.2	0.62	86.0	6.7	1.49	96.8	2.2	0.86	15.8	4.3	1.20
	8	97.8	2.5	0.91	86.5	11.7	1.98	96.8	2.5	0.91	15.0	3.6	1.09

2	1	99.3	1.0	0.56	92.8	1.0	0.56	95.3	2.5	0.91	70.3	1.7	0.75
	2	99.3	1.0	0.56	90.3	4.1	1.17	92.8	2.8	0.96	63.0	2.4	0.90
	3	98.0	1.4	0.69	88.5	4.0	1.16	98.0	1.2	0.62	58.3	3.4	1.07
	4	99.3	1.0	0.56	91.5	1.3	0.66	97.0	1.8	0.78	49.3	3.5	1.08
	5	90.0	3.6	1.09	69.0	8.0	1.63	97.5	0.6	0.44	61.3	7.0	1.53
	6	88.0	2.7	0.95	53.3	8.5	1.68	95.8	1.7	0.75	55.0	2.9	0.99
	7	98.0	0.8	0.52	91.5	2.4	0.89	93.5	5.9	1.40	49.3	3.8	1.12
	8	99.0	0.8	0.52	95.3	1.0	0.56	93.3	8.8	1.72	49.8	4.9	1.27
3	1	94.0	2.9	0.99	96.8	1.5	0.71	91.3	3.2	1.03	95.5	2.1	0.83
	2	99.0	0.8	0.52	98.0	1.8	0.78	94.5	2.5	0.92	97.0	1.4	0.69
	3	96.0	3.2	1.03	98.8	1.3	0.65	97.8	1.0	0.56	95.5	1.3	0.66
	4	99.8	0.5	0.41	96.5	2.5	0.92	95.3	1.5	0.71	97.3	1.7	0.75
	5	99.0	1.2	0.62	96.3	1.7	0.75	91.3	2.2	0.86	95.5	4.0	1.16
	6	98.5	1.0	0.58	97.3	1.7	0.75	88.3	3.2	1.03	97.5	1.9	0.80
	7	93.8	3.5	1.08	21.8	7.4	1.57	94.8	1.7	0.75	96.0	1.6	0.74
	8	97.0	1.4	0.69	48.8	3.5	1.08	72.8	48.5	4.02	98.8	1.0	0.56
4	1	98.8	1.0	0.56	92.3	5.2	1.32	88.3	1.5	0.71	78.3	5.6	1.37
	2	98.5	1.3	0.66	84.8	2.9	0.98	84.5	4.4	1.22	87.0	2.4	0.90
	3	98.8	0.5	0.41	85.5	2.6	0.94	84.5	2.6	0.94	84.0	3.9	1.14
	4	97.5	2.1	0.83	87.5	2.1	0.83	83.0	2.2	0.85	84.3	3.6	1.09
	5	98.0	0.8	0.52	87.3	4.5	1.22	84.0	3.6	1.09	85.5	1.7	0.76
	6	94.8	3.4	1.07	82.3	3.6	1.09	79.3	3.9	1.13	85.0	1.8	0.78
	7	93.3	3.5	1.08	81.5	3.4	1.07	86.0	0.8	0.52	87.5	1.3	0.66
	8	97.8	1.3	0.65	80.8	4.2	1.18	86.0	1.4	0.69	85.5	3.4	1.07
72	1	0.3	0.5	0.41	0.3	0.5	0.41	1.0	1.4	0.69	3.0	2.2	0.85
	2	98.8	1.0	0.56	98.0	0.8	0.52	82.0	4.8	1.26	81.3	2.6	0.94
	3	0.0	0.0	0.00	0.0	0.0	0.00	1.0	1.2	0.62	0.5	0.6	0.44
	4	98.3	1.7	0.75	97.0	1.4	0.69	74.5	3.5	1.08	54.5	10.2	1.85
	5	0.8	1.0	0.56	1.0	1.4	0.69	0.8	1.0	0.56	0.3	0.5	0.41
	6	0.0	0.0	0.00	0.0	0.0	0.00	0.0	0.0	0.00	0.0	0.0	0.00
	7	0.3	0.5	0.41	0.5	0.6	0.44	0.3	0.5	0.41	1.0	1.4	0.69
	8	0.0	0.0	0.00	0.0	0.0	0.00	0.0	0.0	0.00	84.0	2.6	0.93
